# No adverse effects of transgenic maize on population dynamics of endophytic *Bacillus subtilis* strain B916‐gfp

**DOI:** 10.1002/mbo3.404

**Published:** 2016-09-25

**Authors:** Chongsi Sun, Lili Geng, Meiling Wang, Gaoxiang Shao, Yongfeng Liu, Changlong Shu, Jie Zhang

**Affiliations:** ^1^ State Key Laboratory for Biology of Plant Diseases and Insect PestsInstitute of Plant ProtectionChinese Academy of Agricultural SciencesBeijingChina; ^2^ Institute of Plant ProtectionJiangsu Academy of Agricultural ScienceNanjingChina

**Keywords:** *Bacillus subtilis* strain B916‐gfp, colonization, *cry1Ah* transgenic maize, endophytic bacteria

## Abstract

Endophytic bacterial communities play a key role in promoting plant growth and combating plant diseases. However, little is known about their population dynamics in plant tissues and bulk soil, especially in transgenic crops. This study investigated the colonization of transgenic maize harboring the *Bacillus thuringiensis* (Bt) *cry1Ah* gene by *Bacillus subtilis* strain B916‐gfp present in plant tissues and soil. Bt and nontransgenic maize were inoculated with B916‐gfp by seed soaking, or root irrigation under both laboratory greenhouse and field conditions. During the growing season, B916‐gfp colonized transgenic as well as nontransgenic plants by both inoculation methods. No differences were observed in B916‐gfp population size between transgenic and nontransgenic plants, except at one or two time points in the roots and stems that did not persist over the examination period. Furthermore, planting transgenic maize did not affect the number of B916‐gfp in bulk soil in either laboratory or field trials. These results indicate that transgenic modification of maize with the *cry1Ah* gene has no influence on colonization by the endophytic bacteria B916‐gfp present in the plant and in bulk soil.

## 1 Introduction

Genetically modified (GM) crops planted from 1996 to 2014 cover 181.5 billion hectares globally (James, 2015). These crops, modified with *Bacillus thuringiensis* (Bt) proteins, reduce pesticide use by 500 million kilograms of active ingredient, and had a market value of $15.7 billion in 2014. Currently, 93% of maize planted worldwide is genetically modified (James, 2015). Despite huge economic benefits, GM crops – especially Bt maize – still trigger controversial debates over biosafety and ecological compatibility, including their effects on soil microbial community structure.

Rhizospheric soil microbia play an important role in plant growth, nutrient accumulation, and resistance to biotic and abiotic stressors (de Zelicourt, Al‐Yousif, & Hirt, 2013; Prischl, Hackl, Pastar, Pfeiffer, & Sessitsch, 2012). Some microbial communities in the rhizosphere might move into roots of plant and become root endophytes which were mainly defined by soil type (Bulgarelli et al., 2012). Microbial communities in the rhizosphere have been investigated in terms of the effects of root exudation changes caused by transgenic modification. Bt crops that release Cry protein into field soil have no obvious impact on soil ecology or the diversity of rhizospheric fungi (Miethling‐Graff, Dockhorn, & Tebbe, 2010), and AMF colonization has not been significantly affected by cultivation of Bt maize during the current season (Cheeke, Darby, Rosenstiel, Bever, & Cruzan, 2014) or in previous years (Cheeke, Pace, Rosenstiel, & Cruzan, 2011; Zeng et al., 2014). However, minor effects on AMF infection capacity have been observed in field‐grown Bt 11 and Bt 176 (Castaldini et al., 2005; Turrini, Sbrana, Nuti, Pietrangeli, & Giovannetti, 2005). These minor differences in AMF community composition are more likely due to soil texture and environmental and seasonal factors than to the inserted transgene (Val, Marín, & Mellado, 2009; Xue, Serohijos, Devare, & Thies, 2011). Although the effects of GM crops on fungal diversity and density have been well studied, few reports have focused on the effects on the endophytic community. Recent studies reported that plants of different species or even different genotypes assembled specific endophytic community (Cotta et al., 2013; Donn, Kirkegaard, Perera, Richardson, & Watt, 2014; Gaiero, McCall, Thompson, Day, & Dunfield, 2013). Two studies have investigated the influence of crops transformed with Bt genes on endophytic bacterial communities, and reported no significant effects (da Silva et al., 2014; Prischl et al., 2012).

*Bacillus subtilis* (Bs) promotes plant growth (Bai, D'Aoust, Smith, & Driscoll, 2002; Zaidi, Usmani, Singh, & Musarrat, 2006) and controls plant diseases (Gajbhiye, Rai, Meshram, & Dongre, 2010; Zeriouh et al., 2011) by secreting various metabolites. Bs strain B‐916 was successfully used to control false smut and sheath blight in rice in the Jiangsu province of China (Liu et al., 2007). As a rhizosphere bacterium, Bs strain B‐916 might expose to Bt toxin secreted by roots of Bt crops. This study investigated the effects of transgenic maize 33–7 harboring the *cry1Ah* gene on the population size of Bs strain B‐916. Bt maize 33–7 is effective in controlling the growth of *Ostrinia furnacalis* larvae under both laboratory and field conditions (Wang et al., 2008). A previous study found no obvious adverse effects on microorganisms in rhizosphere soil (Cui, Shu, Song, Gao, & Zhang, 2011), midgut bacterial structure, or the development of honey bees fed with Bt‐cry1Ah maize in our laboratory (Dai et al., 2012, 2012; Geng et al., 2013; Jiang et al., 2013); however, its effects on endophytic bacteria is unknown.

## 2 Experimental Procedures

### 2.1 Maize cultivars and endophytic bacterial strain

Two maize varieties, 33–7 (transgenic line harboring the *cry1Ah* gene driven by constitutive promoter) and inbred lines X090 (nontransgenic parental line), were provided by the Biotechnology Research Institute of the Chinese Academy of Agricultural Sciences (CAAS). Field experiments were carried out at the Langfang Experiment Station of CAAS, Hebei, China in 2012. *B. subtilis* strain B916‐gfp (Yao, Chen, Zheng, Zhang, & Huang, 2003) was provided by the Institute of Plant Protection of Jiangsu Academy of Agricultural Science.

### 2.2 Cry1Ah protein purification

Cry1Ah protein was extracted from *B. thuringiensis* strain Biot1Ah by alkaline solubilization method and purified as described in Xue et al. (2008). Protoxin was dissolved in 50 mmol/L Na_2_CO_3_ (pH 9.6), and then purified by anion‐exchange chromatography using an AKTA FPLC system. To evaluate the effects of Cry1Ah protein, a growth curve of strain B916‐gfp was investigated by monitoring the OD600 of samples at different time points for 24 hr. This procedure was repeated three times.

### 2.3 Inoculation methods and sampling strategy

For inoculation by seed soaking, X090 and 33–7 maize seeds (*n* = 80 each) were surface‐sterilized by treatment with 2% sodium hypochlorite for 5 min. The seeds were soaked in 30 ml B916‐gfp suspension (10^11^ CFU/ml) on a shaker (120 rpm) at 28°C for 8 hr, then transferred to glass tubes (diameter × height, 5 × 40 cm; three seeds per tube) that were covered with sterilized film, and cultured in a greenhouse. Seedlings were collected at 3, 7, 10, 14, and 17 days after germination.

For inoculation by root irrigation, X090 and 33–7 maize seeds (*n* = 60 each) were grown in plastic pots (80 × 60 × 30 cm). Seedlings were irrigated with a B916‐gfp suspension (10^11^ CFU/ml) 7 days after germination in the greenhouse or 11 days after sowing for the field trial on roots. Seedlings and soil samples were collected 7, 14, 21, 28, and 35 days after inoculation.

### 2.4 Colonization assessment

About 500 mg of roots, stems, and leaves collected from three samples were sterilized for 10 min in 0.2% mercuric chloride, then washed four times with sterile water. Surface‐sterilized tissue was ground after adding 1 ml of phosphate‐buffered saline (pH 7.4). Tissue fluids were diluted 10‐, 100‐, and 1000‐fold and spread on Luria Bertani medium agar plates supplemented with 5 μg/ml chloromycetin, which were cultured at 30°C for 72 hr. The number of clones exhibiting green fluorescence was counted under UV light (366 nm). Plasmids were extracted from the cells and the *gfp* gene was amplified by PCR using the following primers: gfpF, 5′‐TAA GGG GGA AAT CAC ATG AGT AAA GGA GAA GAA‐3′ and gfpR, 5′‐GGG GTA CCA TTA TTT TTG ACA CCA GA‐3′ under the conditions: 94°C for 10 min, followed by 30 cycles of 94°C for 1 min, 56°C for 1 min, and 72°C for 2 min. The plasmids were also digested with the restriction enzymes *Kpn*I and *Sph*I.

Genomic DNA was isolated from plant roots, stems, and leaves. The *gfp* gene was amplified using the primers 640F (5′‐GAC ACA ATC TGC CCT TTC GA‐3′) and 702R (5′‐AAT CCC AGC AGC AGA TAC AGA C‐3′) under same conditions of primer gfpF/gfpR. Reactions were performed in triplicate.

Tissue samples were embedded in tissue‐freezing medium and cut into sections at a thickness of 10 μm on a CM 1850 freezing‐stage microtome (Leica, Jena, Germany). Sections were visualized using a Leica TCS DM IRE2 laser scanning confocal microscope.

### 2.5 Quantitative real‐time PCR

Quantitative real‐time PCR analysis was carried out using a 7500 Real‐Time PCR System (Applied Biosystems) with SYBR Premix Dimer Eraser (Perfect Real Time; Takara Bio, Otsu, Japan) and genomic DNA isolated from maize plant roots, stems, and leaves as the template. The reaction conditions were as follows: 95°C for 15 min, followed by 40 cycles of 95°C for 10 s, 56°C for 20 s, and 72°C for 32 s. Reactions were performed in triplicate. Primers 640F and 702R were used to amplify the *gfp* gene. A five‐step dilution series of the *gfp* gene (ranging from 10^4^ to 10^8^ copies) was used as a template to generate a standard curve.

### 2.6 Data analysis

Quantitative real‐time PCR data were analyzed with the least significant difference multiple comparisons test using SPSS v.13.0 software (SPSS Inc., Chicago, IL).

## 3 Results

### 3.1 Colonization of transgenic maize by Bs strain B916‐green fluorescent protein (gfp) inoculated by seed soaking under greenhouse conditions

**Figure 1:**
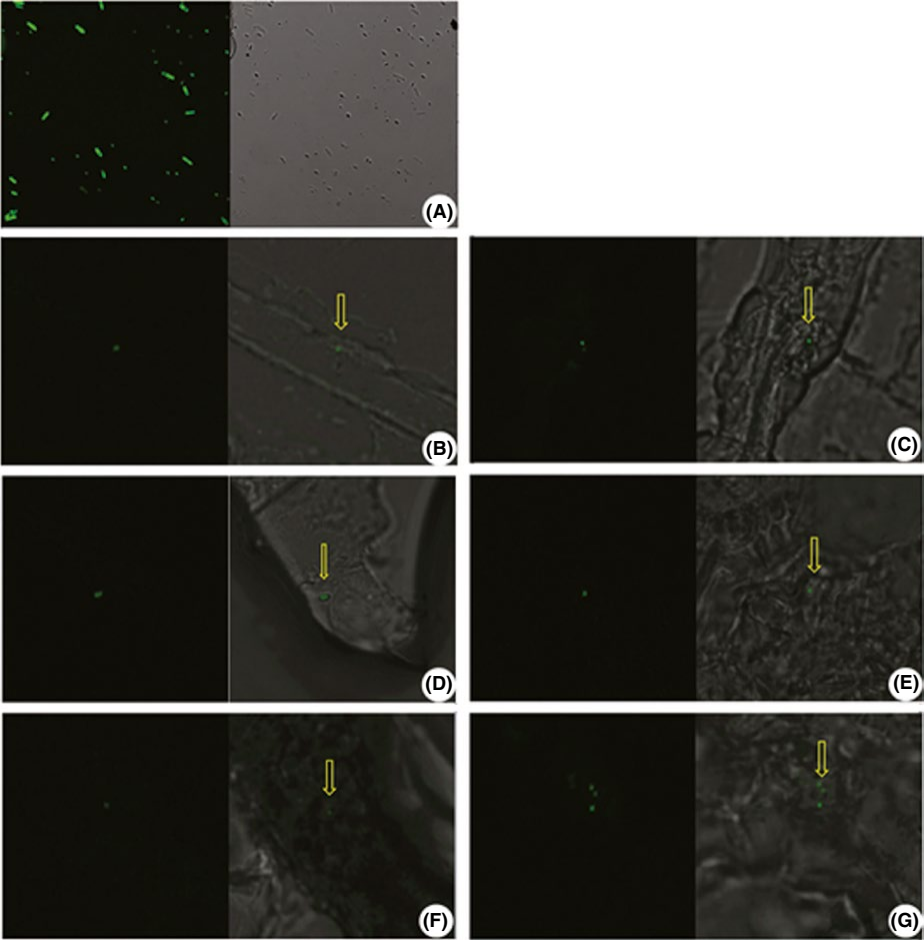
Green fluorescence in Bs B916‐gfp cells. Fluorescent signals (yellow arrows) were detected by confocal microscopy in (A) cells; (B, C) roots; (D, E) stems; and (F, G) leaves of nontransgenic (B, D, F) and transgenic (C, E, G) maize. Left: GFP image. Right: GFP/bright‐field overlay

We first investigated the effects of Cry1Ah protein on the growth of Bs strain B916‐gfp. There were no differences in growth curves of strain B916‐gfp without or with 15, 150, and 300 μg/ml Cry1Ah protein supplementation (Fig. S1). To compare the ability of Bs strain B916‐gfp to colonize transgenic (33–7, harboring the *cry1Ah* gene from Bt) and nontransgenic (X090) maize plants, surface‐sterilized seeds were soaked in a B916‐gfp cell suspension (1011 CFU/ml) for 8 hr before they were grown in a greenhouse. Colonization was verified by confocal microscopy detection of GFP expression and molecular analysis of strain B916‐gfp DNA in the roots, stems, and leaves of each plant. Green fluorescence was observed in all examined parts of transgenic and nontransgenic plants (Fig. 1). After surface sterilization, endophytic bacteria were isolated from maize roots, stems, and leaves and cultured by conventional methods; plasmids were extracted from green fluorescent clones detected under ultraviolet (UV) light (366 nm) (Fig. S2). An 800‐bp fragment was amplified from these clones using *gfp*‐specific primers (Fig. S3A), and 5900‐ and 2300‐bp fragments were obtained by digestion with *Kpn*I and *Sph*I restriction enzymes, as for the positive control plasmid (Fig. S3B).

**Figure 2:**
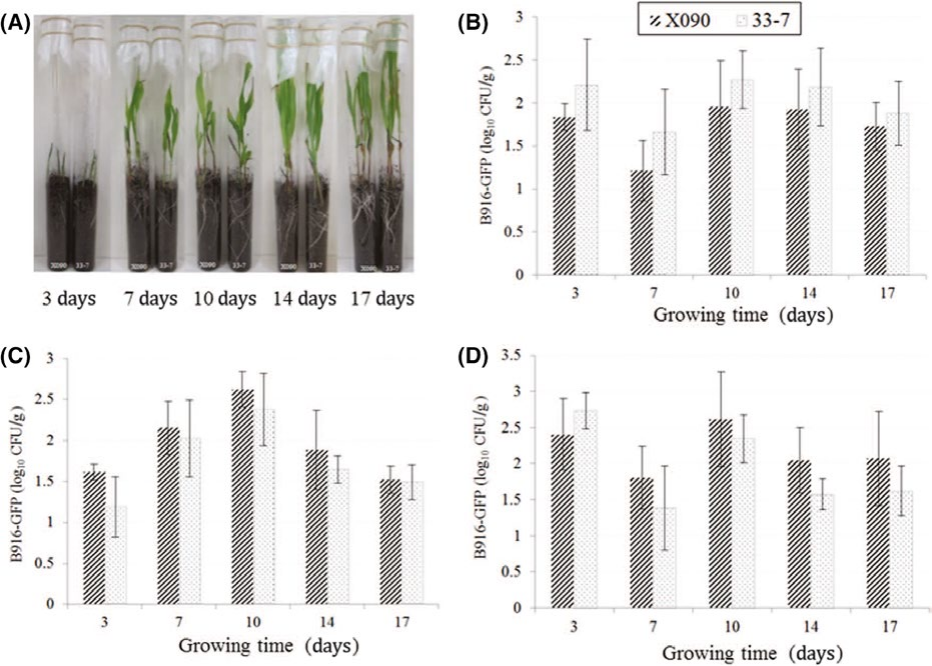
(A) Cultivation of maize by seed‐soaking treatment in a greenhouse. (B–D) Number of B916‐gfp colonies in roots (B), stems (C), and leaves (D) of transgenic and nontransgenic maize plants (*n* = 5)

**Figure 3:**
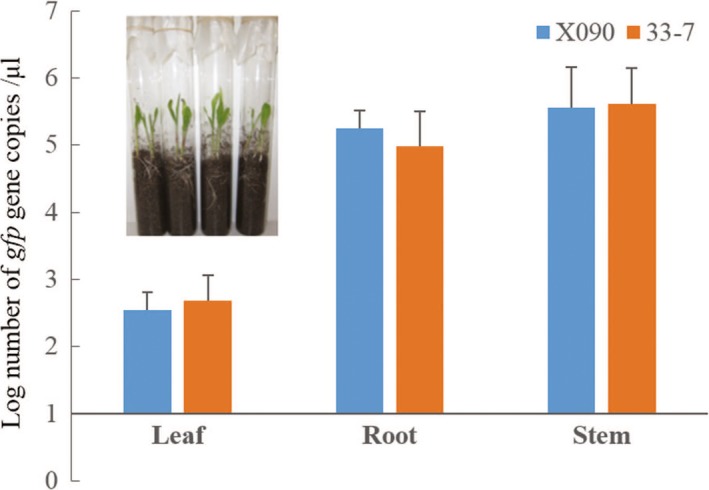
Concentration of the *gfp* gene in roots of transgenic and nontransgenic maize, as determined by quantitative PCR analysis. X090 and 33–7 maize seeds soaked in a B916‐gfp suspension were grown under greenhouse conditions; 150 mg of tissue from each seedling were collected at 5 days (*n* = 5), and total genomic DNA was extracted for quantitative PCR analysis

The number of B916‐gfp cells colonizing roots, stems, and leaves over 17 days' cultivation (Fig. 2A) was counted under UV light (Fig. S2B). B916‐gfp population dynamics were similar in the three tissues in both transgenic and nontransgenic maize, with the number of colonies reaching a peak at 10 days (range: 1.96–2.62 log_10 _CFU/g) before decreasing thereafter (*p* > .05) (Fig. 2B–D). The concentration of the *gfp* gene in the roots, stems, and leaves was comparable for transgenic and nontransgenic maize at 5 days, as determined by quantitative PCR analysis (*p* > .05) (Fig. 3A). Hence, the ability of strain B916‐gfp to colonize maize plants was unaffected by the presence of the transgene.

### 3.2 Colonization of transgenic maize by Bs strain B916‐gfp inoculated by root irrigation under greenhouse and field conditions

Transgenic and nontransgenic maize were root‐irrigated 7 days after seed germination with Bs strain B916‐gfp. Roots, stems, and leaves were collected for analysis every 7 days after inoculation. Under greenhouse conditions, the B916‐gfp population in the roots of transgenic maize reached a maximum value of 2.59 log_10 _CFU/g on day 21 after inoculation (Table 1). In the other tissues of both transgenic and nontransgenic maize, the number of B916‐gfp cells peaked at 2.05–3.17 log_10 _CFU/g on day 14 after inoculation (Table 1). There were no differences in colony numbers in leaves between transgenic and nontransgenic plants from 7 to 35 days after root irrigation (Table 1). The number of B916‐gfp colonies differed significantly in root samples collected on day 14 and stem samples collected on days 14 and 21, but this difference did not endure for the duration of the examination period (Table 1). Under field conditions, the B916‐gfp population in leaves reached the highest values (2.01 log_10 _CFU/g for transgenic and 2.04 log_10 _CFU g^−1^ for nontransgenic maize) 14 or 21 days after inoculation (Table 1) before decreasing, similar to the trend observed under greenhouse conditions. There were no differences in the number of colonies in leaves between transgenic and nontransgenic plants from 7 to 35 days after root irrigation (Table 1), nor were there differences in B916‐gfp population dynamics in the rhizospheres of transgenic and nontransgenic plants under greenhouse and field conditions (Fig. S4A and B).

**Table 1:** Number of B916‐gfp colony in different tissues of maize following root‐irrigation treatment (*n* = 6)

Growing condition	Tissues	Maize variety	log_10 _CFU/g				
			7 (Mean ± SD)	14 (Mean ± SD)	21 (Mean ± SD)	28 (Mean ± SD)	35 (Mean ± SD)
Greenhouse	Roots	X090	1.05 ± 0.49	2.39 ± 0.31*	1.85 ± 0.69	1.71	–
		33–7	1.73 ± 0.22	1.95 ± 0.15*	2.59 ± 0.60	1.81 ± 1.04	1.48 ± 0.47
	Stems	X090	1.94	2.05 ± 0.49*	0.96 ± 0.65*	1.32 ± 0.63	1.03 ± 0.61
		33–7	–	3.18 ± 0.33*	2.64 ± 0.29*	1.11 ± 0.67	–
	Leaves	X090	1.49	2.84 ± 0.43	1.98 ± 0.81	1.10 ± 0.79	–
		33–7	1.26 ± 0.43	2.42 ± 0.05	1.65 ± 0.50	1.45 ± 1.68	–
Field	Leaves	X090	–	1.44 ± 0.16	2.04 ± 1.16	1.12 ± 0.79	0.26 ± 0.11
		33–7	–	2.01 ± 1.35	1.92 ± 0.75	1.25 ± 0.78	–

*p < .05 (analysis of variance).

## 4 Discussion

This laboratory and field study investigated the impact of *cry1Ah* maize on the colonization ability and population size of B916‐gfp endophytic bacteria. We found that planting transgenic *cry1Ah* maize did not affect the size of B916‐gfp populations in the rhizosphere soil, and that the cells could colonize transgenic and nontransgenic maize with equal efficiency by seed‐soaking and root‐irrigation inoculation methods. Moreover, there were no significant differences observed in terms of colony number between transgenic and nontransgenic plants, except for one or two time points in roots and stems that did not persist over the period of examination.

Soil bacteria are exposed to Bt toxins released into rhizosphere soil through root exudates from Bt crops (Gruber, Paul, Meyer, & Müller, 2012; Wang et al., 2013; Xue, Diaz, & Thies, 2014). A recent study reported that desorbed Bt protein was quickly mineralized by microbial degradation (Valldor, Miethling‐Graff, Martens, & Tebbe, 2015). Several studies have investigated the effect of growing Bt maize on the diversity and population size of microbial rhizosphere communities, especially AMF (Castaldini et al., 2005; Cheeke et al., 2011, 2014; Turrini et al., 2005; Zeng et al., 2014); only two examined endophytic bacterial communities, which were not significantly affected by transformation of maize with *cry1Ab* (da Silva et al., 2014), *cry3Bb1*,* cry1A105*, or *cry1Ab2* genes (Prischl et al., 2012). Here, we used Bs strain B916 expressing the GFP marker to monitor the population dynamics and colonization of Bt maize and its parental line. Bs strain B916 was confirmed as an endophytic bacteria based on the green fluorescence detected in the roots, stems, and leaves of plants and by PCR amplification of the *gfp* gene. B916‐gfp cells were transferred from the outside to the inside of maize plant tissues by inoculation of seeds and roots; they also moved from the inoculated parts of the plant to other parts such as the leaves and stem following root irrigation. So, Bs strain B916 was exposed to Cry1Ah protein in the tissues of maize as well as in the soil. There were no significant differences in the population dynamics of B916‐gfp colonies in transgenic and nontransgenic maize under laboratory or field conditions. This was consistent with the colonization of AMF (Cheeke et al., 2011, 2014; Zeng et al., 2014) and endophytic bacterial communities (da Silva et al., 2014; Prischl et al., 2012) in Bt maize. After inoculation, the growth curve of B916‐gfp followed the same trend of first increasing then subsequently declining in tissues of *cry1Ah* maize and isogenic lines. In leaves collected 14 days after root‐irrigation treatment, the number of B916‐gfp cells was about 2.5 log_10 _CFU/g in the laboratory trial as compared to 2.0 log_10 _CFU/g in the field trial. This difference was likely due to temperature, humidity, light intensity, and soil texture.

In conclusion, we found that B916 is an endophytic bacteria that is translocated from the roots of maize plants to the stem and leaves. There were no significant differences in population size or dynamics between B916‐gfp colonies in transgenic and nontransgenic plants in either laboratory or field conditions. Since endophytic bacteria can hasten growth of plants, improve resistance to diseases and environmental stress, this study will provide a new insight into biosafety analysis of GM crops.

## Conflict of Interest

None declared.

## References

[mbo3404-bib-0001] Bai, Y. , D'Aoust, F. , Smith, D. L. , & Driscoll, B. T. (2002). Isolation of plant‐growth‐promoting *Bacillus* strains from soybean root nodules. Canadian Journal of Microbiology, 48, 230–238. 11989767 10.1139/w02-014

[mbo3404-bib-0101] Bulgarelli, D. , Rott, M. , Schlaeppi, K. , Ver Loren van Themaat, E. , Ahmadinejad, N. , Assenza, F. , … Schulze‐Lefert, P. (2012). Revealing structure and assembly cues for Arabidopsis root‐inhabiting bacterial microbiota. Nature, 488, 91–95. 22859207 10.1038/nature11336

[mbo3404-bib-0002] Castaldini, M. , Turrini, A. , Sbrana, C. , Benedetti, A. , Marchionni, M. , Mocali, S. , … Giovannetti, M. (2005). Impact of Bt corn on rhizospheric and soil eubacterial communities and on beneficial mycorrhizal symbiosis in experimental microcosms. Applied and Environment Microbiology, 71, 6719–6729. 10.1128/AEM.71.11.6719-6729.2005 PMC1287690 16269702

[mbo3404-bib-0003] Cheeke, T. E. , Darby, H. , Rosenstiel, T. N. , Bever, J. D. , & Cruzan, M. B. (2014). Effect of *Bacillus thuringiensis* (Bt) maize cultivation history on arbuscular mycorrhizal fungal colonization, spore abundance and diversity, and plant growth. Agriculture, Ecosystems & Environment, 195, 29–35.

[mbo3404-bib-0004] Cheeke, T. E. , Pace, B. A. , Rosenstiel, T. N. , & Cruzan, M. B. (2011). The influence of fertilizer level and spore density on arbuscular mycorrhizal colonization of transgenic Bt 11 maize (*Zea mays*) in experimental microcosms. FEMS Microbiology Ecology, 75, 304–312. 21198682 10.1111/j.1574-6941.2010.01013.x

[mbo3404-bib-0005] Cotta, S. R. , Dias, A. C. F. , Marriel, I. E. , Gomes, E. A. , Van Elsas, J. D. , & Seldin, L. (2013). Temporal dynamics of microbial communities in the rhizosphere of two genetically modified (GM) maize hybrids in tropical agrosystems. Antonie van Leeuwenhoek, 103, 589–601. 23124960 10.1007/s10482-012-9843-7

[mbo3404-bib-0006] Cui, H. , Shu, C. , Song, F. , Gao, J. , & Zhang, J. (2011). Effect of cry1Ah‐transgenic maize on community structure of microorganism in rhizosphere soil. Journal of Northeast Agricultural University, 42, 30–38.

[mbo3404-bib-0007] da Silva, D. A. F. , Cotta, S. R. , Vollú, R. E. , Jurelevicius, D. A. , Marques, J. M. , Marriel, I. E. , & Seldin, L. (2014). Endophytic microbial community in two transgenic maize genotypes and in their near‐isogenic non‐transgenic maize genotype. BMC Microbiology, 14, 332. 25540019 10.1186/s12866-014-0332-1 PMC4327796

[mbo3404-bib-0008] Dai, P. , Zhou, W. , Zhang, J. , Cui, H. , Wang, Q. , Jiang, W. , … Zhou, T. (2012). Field assessment of Bt *cry1Ah* corn pollen on the survival, development and behavior of *Apis mellifera ligustica* . Ecotoxicology and Environmental Safety, 79, 232–237. 22364780 10.1016/j.ecoenv.2012.01.005

[mbo3404-bib-0009] Dai, P. , Zhou, W. , Zhang, J. , Jiang, W. , Wang, Q. , Cui, H. , … Zhou, T. (2012). The effects of Bt *Cry1Ah* toxin on worker honeybees (*Apis mellifera ligustica* and *Apis cerana cerana*). Apidologie, 43, 384–391.

[mbo3404-bib-0010] de Zelicourt, A. , Al‐Yousif, M. , & Hirt, H. (2013). Rhizosphere microbes as essential partners for plant stress tolerance. Molecular Plant, 6, 242–245. 23475999 10.1093/mp/sst028

[mbo3404-bib-0011] Donn, S. , Kirkegaard, J. A. , Perera, G. , Richardson, A. E. , & Watt, M. (2014). Evolution of bacterial communities in the wheat crop rhizosphere. Environmental Microbiology, 17, 610–621. 24628845 10.1111/1462-2920.12452

[mbo3404-bib-0012] Gaiero, J. R. , McCall, C. A. , Thompson, K. A. , Day, N. J. , & Dunfield, K. E. (2013). Inside the root microbiome: Bacterial root endophytes and plant growth promotion. American Journal of Botany, 100, 1738–1750. 23935113 10.3732/ajb.1200572

[mbo3404-bib-0013] Gajbhiye, A. , Rai, A. R. , Meshram, S. U. , & Dongre, A. (2010). Isolation, evaluation and characterization of *Bacillus subtilis* from cotton rhizospheric soil with biocontrol activity against *Fusarium oxysporum* . World Journal of Microbiology & Biotechnology, 26, 1187–1194. 24026922 10.1007/s11274-009-0287-9

[mbo3404-bib-0014] Geng, L. , Cui, H. , Dai, P. , Lang, Z. , Shu, C. , Zhou, T. , … Zhang, J. (2013). The influence of Bt‐transgenic maize pollen on the bacterial diversity in the midgut of *Apis mellifera ligustica* . Apidologie, 44, 198–208.

[mbo3404-bib-0015] Gruber, H. , Paul, V. , Meyer, H. D. , & Müller, M. (2012). Determination of insecticidal Cry1Ab protein in soil collected in the final growing seasons of a nine‐year field trial of Bt‐maize MON810. Transgenic Research, 21, 77–88. 21499757 10.1007/s11248-011-9509-7

[mbo3404-bib-0016] James, C. (2015). Global status of commercialized biotech/GM crops: 2013. ISAAA Brief No 49‐2014.

[mbo3404-bib-0017] Jiang, W. , Geng, L. , Dai, P. , Lang, Z. , Shu, C. , Lin, Y. , … Zhang, J. (2013). The influence of Bt‐transgenic maize pollen on the bacterial diversity in the midgut of Chinese honeybees, *Apis cerana cerana* . Journal of Integrative Agriculture, 12, 474–482.

[mbo3404-bib-0018] Liu, Y. , Chen, Z. , Ng, T. B. , Zhang, J. , Zhou, M. , Song, F. , … Liu, Y. (2007). Bacisubin, an antifungal protein with ribonuclease and hemagglutinating activities from *Bacillus subtilis* strain B‐916. Peptides, 28, 553–559. 17129637 10.1016/j.peptides.2006.10.009

[mbo3404-bib-0019] Miethling‐Graff, R. , Dockhorn, S. , & Tebbe, C. C. (2010). Release of the recombinant Cry3Bb1 protein of Bt maize MON88017 into field soil and detection of effects on the diversity of rhizosphere bacteria. European Journal of Soil Biology, 46, 41–48.

[mbo3404-bib-0020] Prischl, M. , Hackl, E. , Pastar, M. , Pfeiffer, S. , & Sessitsch, A. (2012). Genetically modified Bt maize lines containing *cry3Bb1*,* cry1A105* or *cry1Ab2* do not affect the structure and functioning of root‐associated endophyte communities. Applied Soil Ecology, 54, 39–48.

[mbo3404-bib-0021] Turrini, A. , Sbrana, C. , Nuti, M. , Pietrangeli, B. , & Giovannetti, M. (2005). Development of a model system to assess the impact of genetically modified corn and aubergine plants on arbuscular mycorrhizal fungi. Plant and Soil, 266, 69–75.

[mbo3404-bib-0022] Val, G. , Marín, S. , & Mellado, R. (2009). A sensitive method to monitor *Bacillus subtilis* and *Streptomyces coelicolor*‐related bacteria in maize rhizobacterial communities: The use of genome‐wide microarrays. Microbial Ecology, 58, 108–115. 18855043 10.1007/s00248-008-9451-2

[mbo3404-bib-0023] Valldor, P. , Miethling‐Graff, R. , Martens, R. , & Tebbe, C. (2015). Fate of the insecticidal Cry1Ab protein of GM crops in two agricultural soils as revealed by 14C‐tracer studies. Applied Microbiology and Biotechnology, 99, 7333–7341. 25967657 10.1007/s00253-015-6655-5

[mbo3404-bib-0024] Wang, Y. , Hu, H. , Huang, J. , Li, J. , Liu, B. , & Zhang, G. (2013). Determination of the movement and persistence of Cry1Ab/1Ac protein released from Bt transgenic rice under field and hydroponic conditions. Soil Biology & Biochemistry, 58, 107–114.

[mbo3404-bib-0025] Wang, Y. , Lang, Z. , Zhang, J. , He, K. , Song, F. , & Huang, D. (2008). Ubi1 intron‐mediated enhancement of the expression of Bt *cry1Ah* gene in transgenic maize (*Zea mays* L.). Chinese Science Bulletin, 53, 3185–3190.

[mbo3404-bib-0026] Xue, K. , Diaz, B. R. , & Thies, J. E. (2014). Stability of Cry3Bb1 protein in soils and its degradation in transgenic corn residues. Soil Biology & Biochemistry, 76, 119–126.

[mbo3404-bib-0027] Xue, J. , Liang, G. , Crickmore, N. , Li, H. , He, K. , Song, F. , Feng, X. , Huang, D. , & Zhang, J. (2008). Cloning and characterization of a novel Cry1A toxin from *Bacillus thuringiensis* with high toxicity to the Asian corn borer and other lepidopteran insects. FEMS Microbiology Letters, 280, 95–101. 18248430 10.1111/j.1574-6968.2007.01053.x

[mbo3404-bib-0028] Xue, K. , Serohijos, R. C. , Devare, M. , & Thies, J. E. (2011). Decomposition rates and residue‐colonizing microbial communities of *Bacillus thuringiensis* insecticidal protein Cry3Bb‐expressing (Bt) and Non‐Bt corn hybrids in the field. Applied and Environment Microbiology, 77, 839–846. 10.1128/AEM.01954-10 PMC3028697 21148693

[mbo3404-bib-0029] Yao, Z. S. , Chen, Z. Y. , Zheng, X. B. , Zhang, J. , & Huang, D. F. (2003). Genetically marking of natural biocontrol bacterium *Bacillus subtilis* strains with green fluorescent protein gene. Chinese Journal of Biotechnology, 19, 551–555. 15969082

[mbo3404-bib-0030] Zaidi, S. , Usmani, S. , Singh, B. R. , & Musarrat, J. (2006). Significance of *Bacillus subtilis* strain SJ‐101 as a bioinoculant for concurrent plant growth promotion and nickel accumulation in *Brassica juncea* . Chemosphere, 64, 991–997. 16487570 10.1016/j.chemosphere.2005.12.057

[mbo3404-bib-0031] Zeng, H. , Tan, F. , Zhang, Y. , Feng, Y. , Shu, Y. , & Wang, J. (2014). Effects of cultivation and return of *Bacillus thuringiensis* (Bt) maize on the diversity of the arbuscular mycorrhizal community in soils and roots of subsequently cultivated conventional maize. Soil Biology & Biochemistry, 75, 254–263.

[mbo3404-bib-0032] Zeriouh, H. , Romero, D. , García‐Gutiérrez, L. , Cazorla, F. M. , de Vicente, A. , & Pérez‐García, A. (2011). The iturin‐like lipopeptides are essential components in the biological control arsenal of *Bacillus subtilis* against bacterial diseases of cucurbits. Molecular Plant‐Microbe Interactions, 24, 1540–1552. 22066902 10.1094/MPMI-06-11-0162

