# Supplementary figures and images for: No adverse effects of transgenic maize on population dynamics of endophytic Bacillus subtilis strain B916‐gfp
Source: Microbiologyopen. 2016 Sep 25;6(1):e00404. doi: 10.1002/mbo3.404 (PMC5300882; doi:10.1002/mbo3.404)

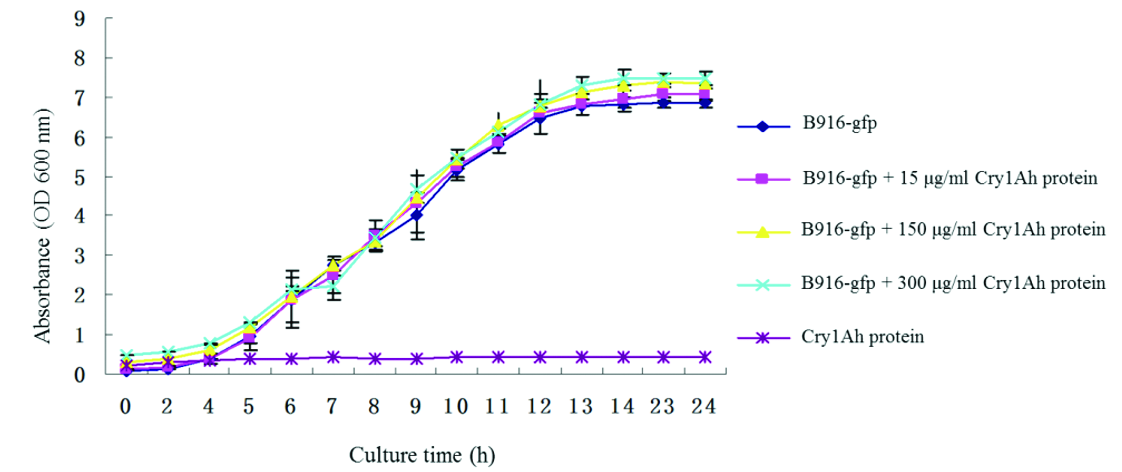

Supplement: Supplementary file 1 [file MBO3-6-0-s001.tif]

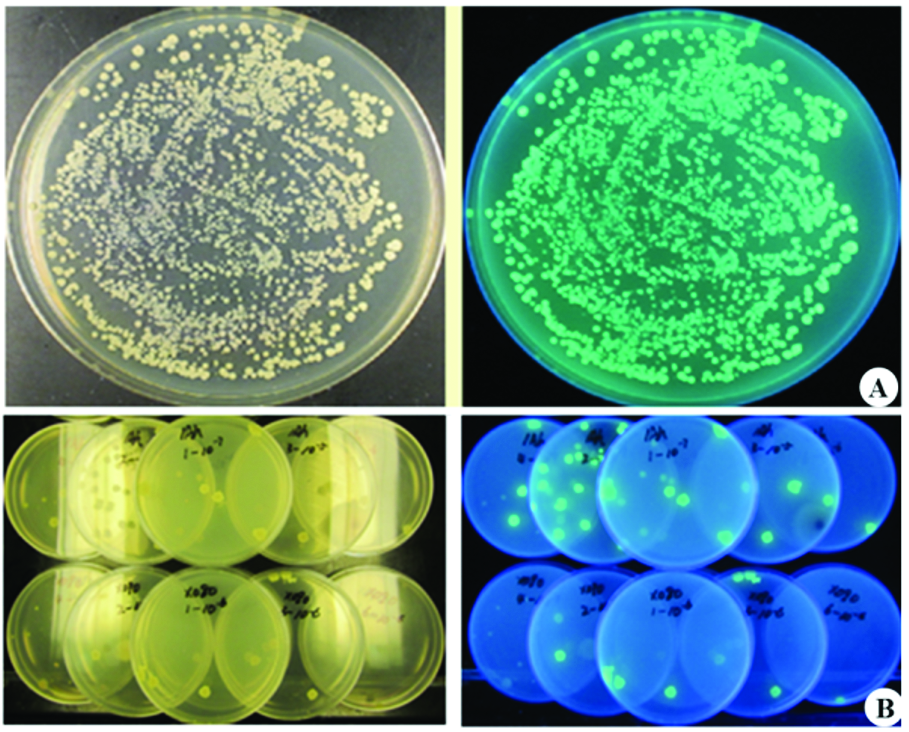

Supplement: Supplementary file 2 [file MBO3-6-0-s002.tif]

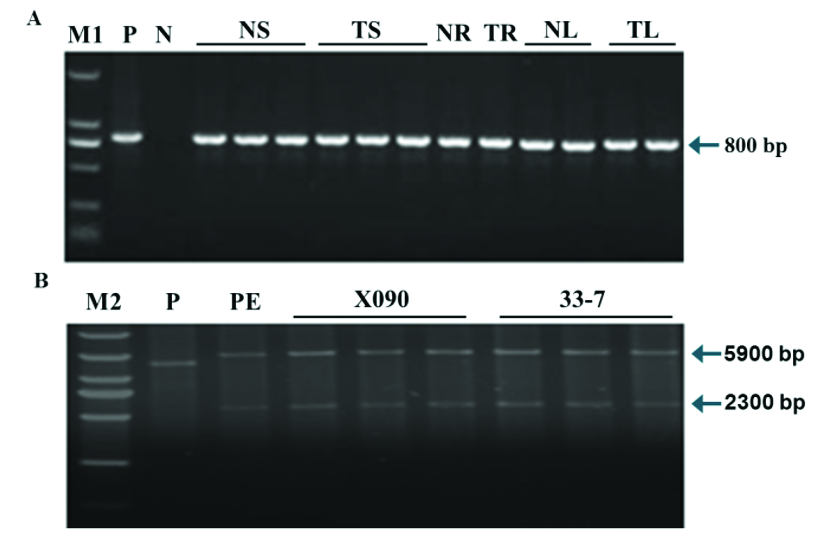

Supplement: Supplementary file 3 [file MBO3-6-0-s003.tif]

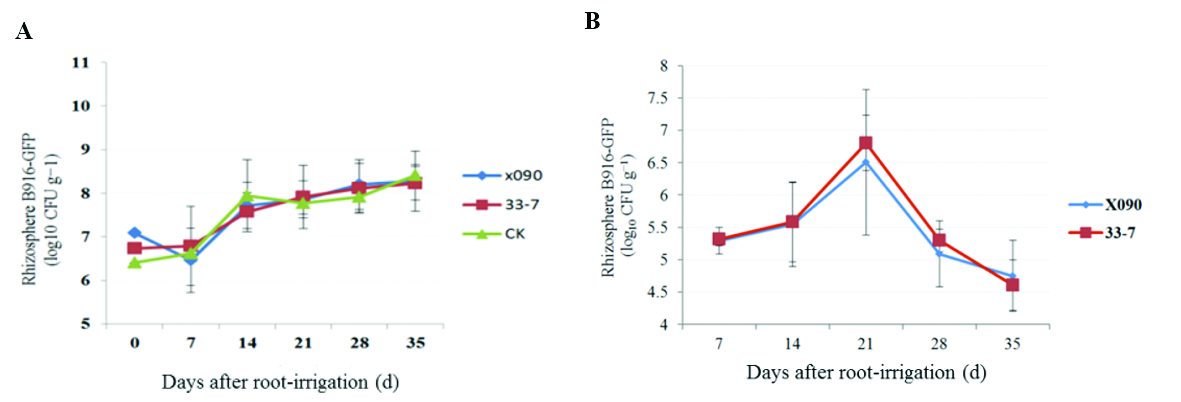

Supplement: Supplementary file 4 [file MBO3-6-0-s004.tif]
